# Automated Identification of Aspirin-Exacerbated Respiratory Disease Using Natural Language Processing and Machine Learning: Algorithm Development and Evaluation Study

**DOI:** 10.2196/44191

**Published:** 2023-06-12

**Authors:** Thanai Pongdee, Nicholas B Larson, Rohit Divekar, Suzette J Bielinski, Hongfang Liu, Sungrim Moon

**Affiliations:** 1 Division of Allergic Diseases Mayo Clinic Rochester, MN United States; 2 Division of Clinical Trials and Biostatistics Department of Quantitative Health Sciences Mayo Clinic Rochester, MN United States; 3 Division of Epidemiology Department of Quantitative Health Sciences Mayo Clinic Rochester, MN United States; 4 Department of Artificial Intelligence and Informatics Mayo Clinic Rochester, MN United States

**Keywords:** aspirin exacerbated respiratory disease, natural language processing, electronic health record, identification, machine learning, aspirin, asthma, respiratory illness, artificial intelligence, natural language processing algorithm

## Abstract

**Background:**

Aspirin-exacerbated respiratory disease (AERD) is an acquired inflammatory condition characterized by the presence of asthma, chronic rhinosinusitis with nasal polyposis, and respiratory hypersensitivity reactions on ingestion of aspirin or other nonsteroidal anti-inflammatory drugs (NSAIDs). Despite AERD having a classic constellation of symptoms, the diagnosis is often overlooked, with an average of greater than 10 years between the onset of symptoms and diagnosis of AERD. Without a diagnosis, individuals will lack opportunities to receive effective treatments, such as aspirin desensitization or biologic medications.

**Objective:**

Our aim was to develop a combined algorithm that integrates both natural language processing (NLP) and machine learning (ML) techniques to identify patients with AERD from an electronic health record (EHR).

**Methods:**

A rule-based decision tree algorithm incorporating NLP-based features was developed using clinical documents from the EHR at Mayo Clinic. From clinical notes, using NLP techniques, 7 features were extracted that included the following: AERD, asthma, NSAID allergy, nasal polyps, chronic sinusitis, elevated urine leukotriene E4 level, and documented no-NSAID allergy. MedTagger was used to extract these 7 features from the unstructured clinical text given a set of keywords and patterns based on the chart review of 2 allergy and immunology experts for AERD. The status of each extracted feature was quantified by assigning the frequency of its occurrence in clinical documents per subject. We optimized the decision tree classifier’s hyperparameters cutoff threshold on the training set to determine the representative feature combination to discriminate AERD. We then evaluated the resulting model on the test set.

**Results:**

The AERD algorithm, which combines NLP and ML techniques, achieved an area under the receiver operating characteristic curve score, sensitivity, and specificity of 0.86 (95% CI 0.78-0.94), 80.00 (95% CI 70.82-87.33), and 88.00 (95% CI 79.98-93.64) for the test set, respectively.

**Conclusions:**

We developed a promising AERD algorithm that needs further refinement to improve AERD diagnosis. Continued development of NLP and ML technologies has the potential to reduce diagnostic delays for AERD and improve the health of our patients.

## Introduction

Aspirin-exacerbated respiratory disease (AERD) is an acquired inflammatory condition characterized by the presence of asthma, chronic rhinosinusitis with nasal polyposis, and respiratory hypersensitivity reactions on ingestion of aspirin or other nonsteroidal anti-inflammatory drugs (NSAIDs) [1]. These reactions typically involve the upper and lower airways and may include nasal congestion, sneezing, rhinorrhea, cough, and wheezing [1]. The prevalence of AERD is approximately 0.3%-0.9% in the general population, but the actual prevalence is unknown in practice, as AERD has no unique International Classification of Diseases, Ninth Revision (ICD-9) or ICD-10 codes [2,3]. In the general population, the mean age of onset of AERD is approximately 30 years [2,4], and the prevalence of AERD is estimated to be 7%-15% in individuals with asthma and 10%-16% in individuals with chronic rhinosinusitis with nasal polyposis [5]. Individuals with AERD have significant symptom burden and morbidity, including severe and recalcitrant sinus disease, high rates of polyp recurrence and revision surgery, and higher asthma exacerbation and hospitalization rates [1]. Despite AERD having a classic constellation of symptoms, the diagnosis is often overlooked, with an average of greater than 10 years between the onset of symptoms and diagnosis of AERD [6]. Without a diagnosis, individuals will lack opportunities to receive effective treatments, such as aspirin desensitization or biologic medications [5,7].

One opportunity to improve diagnostic delays with AERD involves leveraging the immense volume of clinical data available in electronic health records (EHRs). By leveraging natural language processing (NLP) and machine learning (ML), analyses of medical concepts from unstructured clinical documents may aid in early detection of AERD [8]. In this study, we developed a combined algorithm of NLP with ML to identify individuals with AERD.

## Methods

### Ethical Considerations

This study was approved by the Mayo Clinic institutional review board as exempted from ethics approval in accordance with the ethical standards of the responsible committee on human experimentation and the Helsinki Declaration of 1975, as revised in 2000.

### Procedure

Patients who were evaluated within the Allergy and Immunology divisions at Mayo Clinic from January 2001 to March 2022 and met diagnostic criteria for AERD based on accepted guidelines [1] were retrospectively identified by chart review. In total, 200 patients with AERD and 200 patients without AERD were identified. Of these patients, we randomly selected 100 patients with AERD and 100 without AERD to serve as the training set, and the remaining were used for the test set.

A rule-based decision tree algorithm incorporating NLP-based features was developed to identify patients with AERD using clinical documents from the EHR at Mayo Clinic. From clinical notes, 7 features were extracted using NLP techniques based on common characteristics of AERD [1]. These features included the following: prior AERD diagnosis, asthma, NSAID allergy, nasal polyps, chronic sinusitis, elevated urine leukotriene E4 level, and documented no-NSAID allergy. “Prior AERD diagnosis” was defined as whether the patient had a diagnosis of AERD before or had suspicion of a high chance of AERD by the physician. For “asthma,” “nasal polyps,” and “chronic sinusitis,” the patient needed to have a diagnosis confirmation by the physician in the clinical documents. “Elevated urine leukotriene E4 level” indicated if the patient had any record in lab results of a urine leukotriene E4 level greater than 104 pg/mg creatinine. “NSAID allergy” was defined as a patient having had a respiratory reaction to an NSAID. Meanwhile, “documented no-NSAID allergy” indicated that a health care provider recorded “unconfirmed or no specific history of NSAID allergy up to date” in the clinical documents. Given the successful use cases of MedTagger [9] to identify disease in different clinical domains [10,11], we used MedTagger to extract these features with the given set of keywords (including typos, abbreviations, and acronyms) and patterns based on the chart review of 2 allergy and immunology experts for AERD. If the extracted features were located in particular note sections (ie, “History of Present Illness,” “Allergies,” “Past Medical/Surgical History,” “Impression/Report/Plan,” “Diagnosis,” “Principal Diagnosis,” “Secondary Diagnoses,” and “Post Procedure Diagnosis”), they were considered valid AERD features. We collected each feature in all clinical documents per patient in the past 5 years from the last clinic visit because clinical characteristics of AERD can evolve over time (ie, development of NSAID allergy).

We counted the number of times each extracted feature appeared in the clinical documents for each patient and used this count as the numerical representation of each feature. To identify the most practical combination of features for discriminating between different presentations of AERD, we optimized the hyperparameters of the classification and regression tree (CART) decision tree classifier with the identified features on the training set using sklearn [12,13]. We performed hyperparameter tuning on 5 different parameters with 1 model setting, as follows: (1) criterion, with options of gini or entropy; (2) maximum depth, ranging from 1 to 10 with an interval of 1; (3) minimum samples split, ranging from 2 to 10 with an interval of 2; (4) minimum samples leaf, ranging from 1 to 10 with an interval of 1; (5) maximum features, ranging from 1 to 7 with an interval of 1; and (6) a fixed random number generation seed was used to ensure reproducibility. Furthermore, to achieve the highest area under the receiver operating characteristics curve (AUC) score, these hyperparameters were tuned for two types of feature sets: (1) quantitatively represented as numerical values per patient and (2) binary, where “1” denotes the presence and “0” denotes the absence or missing status of each extracted feature per patient. We constructed a decision tree using the best feature set with optimized hyperparameters and then calculated the AUC scores for a range of cutoff thresholds from 0.1 to 1.0 in intervals of 0.1 to determine the optimal cutoff threshold based on a given training set. The resulting tree with the optimized parameters and cutoff threshold converted into sequential rule sets to evaluate the performance in the test set.

## Results

In our cohort, the mean age of the 400 patients was 55.5 years, and 54% (216/400) were female. Table 1 displays the descriptive statistics for each feature, comparing the presence or absence of the feature in the training and test sets. Based on the training set, we obtained the sequential rule sets through the optimized decision tree (with criterion as gini, maximum depth as 7, minimum samples leaf as 7, minimum samples split as 2, maximum features as 3, random state as 20, and best cutoff threshold as 0.6 for parameter settings) using the numerical represented feature set in Table 2. The sequential rules listed in Table 2 describe several clinical factors that include diagnosis of AERD (referred to as AERD), diagnosis of allergy to an NSAID (referred to as NSAID allergy), diagnosis of chronic sinusitis, documented history of tolerance to an NSAID (referred to as non-NSAID allergy), and a prior abnormally elevated urine leukotriene E4 level (referred to as LAB).

In Table 2, it was observed that the derived sequential rule, ranging from 1 to 9, captured 28% (56/200) of the cases in the test set. However, a significant portion of the test set (112/200, 56%) was not identified according to the original intended sequential rule but rather by a different sequence rule. For example, rule 6 failed to capture 73 cases, whereas rule 9—which is less strict than rule 6—captured 59 of those 73 cases that were supposed to belong to rule 6. Similarly, rule 3 captured 15 cases of the remaining 18 cases that should have been identified by rule 1. Therefore, the overall accuracy was 0.84.

**Figure 1:**
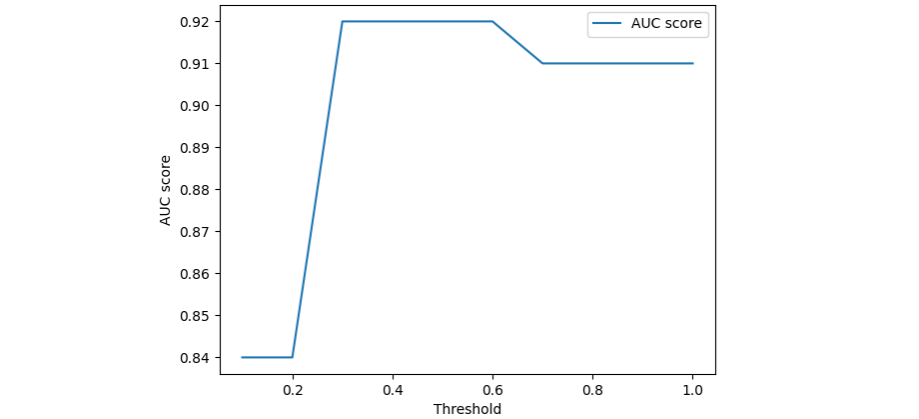
Area under the receiver operating characteristic curve (AUC) scores at different threshold values on the training set.

**Figure 2:**
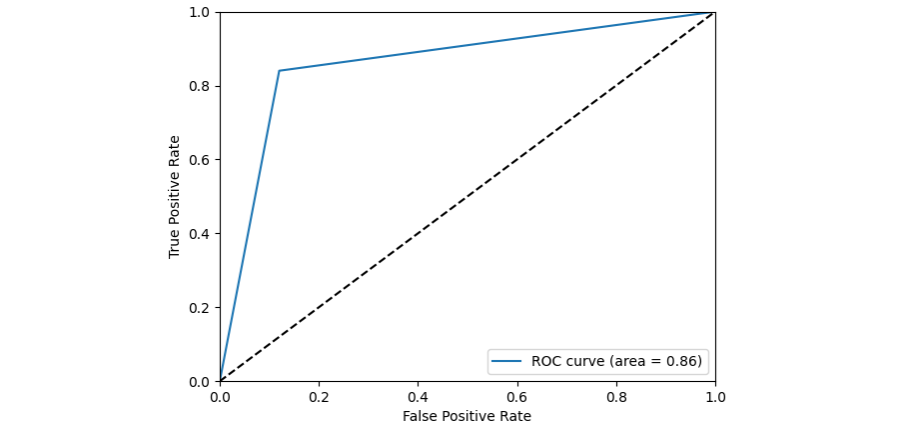
Receiver operating characteristic (ROC) on the test set.

The AERD algorithm achieved an AUC score of 0.92 (95% CI 0.93-1.00) and 0.86 (95% CI 0.78-0.94) for the training and test sets (Figure 1 and Figure 2), respectively. The optimal cutoff point was 0.6 on the training set (Figure 1). Additional performances are presented in Table 3.

**Table 1:** Descriptive statistics of aspirin-exacerbated respiratory disease (AERD) features, describing its presence as 1 and absence as 0 (N=200).

AERD Feature	Train, n (%)	Test, n (%)
AERD	103 (52)	60 (30)
Asthma	192 (96)	82 (41)
NSAID^a^ allergy	98 (49)	121 (61)
Nasal polyps	175 (88)	192 (96)
Chronic sinusitis	182 (91)	180 (90)
LAB^b^	93 (47)	179 (90)
Documented no-NSAID allergy	70 (35)	101 (51)

^a^NSAID: nonsteroidal anti-inflammatory drug.

^b^LAB refers to the elevated urine leukotriene E4 level.

**Table 2:** Derived sequential rules for aspirin-exacerbated respiratory disease (AERD) algorithm and the resulting performance in the test set.

Rule	Sequential rules	AERD	Case, n	Correct, n	Error, n	Confidence (%)^a^
1	AERD≤3.5, NSAID allergy^b^≤2.5, Chronic Sinusitis^c^≤6.5, and then documented non-NSAID allergy≤0.5	No	30	12	0	40
2	AERD≤3.5, NSAID allergy≤2.5, Chronic Sinusitis≤6.5, and then documented non-NSAID allergy>0.5	No	9	0	0	0
3	AERD≤3.5, NSAID allergy≤2.5, Chronic Sinusitis>6.5, and then documented non-NSAID allergy≤0.5	No	43	40	0	93
4	AERD≤3.5, NSAID allergy≤2.5, Chronic Sinusitis>6.5, and then documented non-NSAID allergy>0.5	No	4	3	0	75
5	AERD≤3.5, NSAID allergy>2.5, and then Chronic Sinusitis≤9.0	Yes	10	0	0	0
6	AERD≤3.5, NSAID allergy>2.5, and then Chronic Sinusitis>9.0	Yes	74	1	0	1
7	AERD>3.5, NSAID allergy≤1.5, and then LAB^d^≤0.5	Yes	0	0	0	0
8	AERD>3.5, NSAID allergy≤1.5, and then LAB>0.5	No	2	0	0	0
9	AERD>3.5, NSAID allergy>1.5	Yes	2	0	0	0
10	Others	Yes	16	0	0	0
		No	10	0	0	0
N/A^e^	The cases were not identified according to the original intended sequential rule; instead, a different sequence rule was used.	Yes	99	79	20	80
		No	45	33	12	73

^a^Confidence = the numbers of correct cases divided by numbers of real cases in the test set multiplied by 100 for the particular rule from 1 to 9.

^b^NSAID allergy refers to diagnosis of allergy to a nonsteroidal anti-inflammatory drug (NSAID).

^c^Chronic sinusitis refers to diagnosis of chronic sinusitis.

^d^LAB refers to a prior abnormally elevated urine leukotriene E4 level.

^e^N/A: not applicable.

**Table 3:** Performance of the rule-based aspirin-exacerbated respiratory disease (AERD) algorithm.

Data set	Sensitivity (%; 95% CI)	Specificity (%; 95% CI)	Positive predictive value (%; 95% CI)	Negative predictive value (%; 95% CI)	Accuracy (%; 95% CI)
Train	88.00 (79.98-93.64)	97.00 (91.48-99.38)	96.70 (90.67-99.31)	88.99 (81.56-94.18)	92.50 (87.93-95.74)
Test	80.00 (70.82-87.33)	88.00 (79.98-93.640	86.96 (78.32-93.07)	81.48 (72.86-88.31)	84.00 (78.17-88.79)

## Discussion

### Principal Findings

In our study, we demonstrated that an algorithm, which combines NLP and ML techniques, can identify patients with AERD with a positive predictive value of approximately 86.96 and a negative predictive value of 81.48. Our results are comparable to prior work [3] on automated diagnosis of AERD from EHR data using structured query language statements for data analysis and resulting in positive predictive values ranging from 78.4 to 88.7, depending on the cohort being analyzed.

Prior diagnosis of AERD presents the highest impacted feature (ie, a majority of sequential rules contain prior diagnosis of AERD feature) to detect diagnosis of AERD. In the training and test sets, 85% (85/100) and 91% (91/100) of patients with AERD had a prior diagnosis of AERD, respectively. We also extracted new clinical factors associated with AERD (“elevated urine leukotriene E4 level” and “alcohol intolerance”) that were not previously studied. Furthermore, the “elevated urine leukotriene E4 level” feature may need to be considered as a new meaningful feature associated with AERD because the presence of the term “AERD” with an “elevated leukotriene E4 level” was a common feature of rule sets 7 and 8. Most patients with AERD in the test set were accurately identified by having had an AERD diagnosis and a documented NSAID allergy (Table 2). Lastly, diagnosis of nasal polyps was not used to construct the optimal decision tree, which may indicate that it may be an insignificant feature to distinguish patients with AERD from possible AERD candidates.

The test set included 32 errors from 200 patients, which upon review, were due to either unidentified rule sets for patients with AERD (n=11) or missing and incorrect feature extraction because of unseen keywords or patterns for features (n=9) primarily. For example, the sentence “Patient took an aspirin approximately ten years ago for headache and developed a sensation of pressure in his nose and sinuses” is an unseen pattern for prior AERD features. Based on the expression, “a sensation of pressure in his nose and sinuses,” the sentence should be a prior AERD feature; however, AERD algorithm categorized it as absence of an AERD feature because this pattern was not available in the training phrase. A total of 6 patients had necessary feature information beyond the past 5 years of clinical documents from the last visit day; 6 patients had necessary information belonging to an unknown note section in the training set for feature extraction. When examining the specific rules, rule sets 2-3 resulted in very few errors (Table 2). In contrast, the absence of terms explicitly documenting the absence of NSAID allergy and lack of references to an elevated leukotriene E4 level resulted in more errors in the AERD algorithm.

Diagnosing and confirming AERD may be a prolonged process, as the associated clinical features may present at different times in a variety of time sequences. As a result, there is no solid ICD code (structured data) to represent AERD, and AERD-associated clinical characteristics are often undocumented in clinical texts (unstructured data) in the EHR. This lack of information regarding AERD results in the low quality of data sources and potential bias for ML models [14]. Additional efforts (eg, standardizing routine exams for AERD) are necessary to fill these missing information gaps in practice.

This AERD algorithm has limitations in deploying to detect patients with confirmed AERD in a practical setting without further refinement. We focused on identifying feature selections in the limited parameter tuning using a balanced data set (N=200 for patients with AERD and N=200 for patients without AERD), which was not a real-world situation. We used the minimum sample size due to the nature of AERD, which has a low prevalence. The rule-based algorithm is used because the limited sample and feature set provide high interpretability and accuracy at downstream tasks rather than neural network MLs, which require a large training data set. However, this algorithm provides a valuable contribution to capturing potential patients with AERD in the setting of a large health system EHR because the prevalence of patients with AERD is low in clinical settings. To follow up, we plan to rank features with diverse identified feature sets and parameter tuning for the decision tree model within a large cohort. We will investigate our new feature in the EHR, which is information about urine leukotriene E4 levels in the extensive feature selections, and we will explore additional features for AERD (eg, alcohol sensitivity, anosmia, and prior sinus surgeries).

### Conclusions

We developed an AERD algorithm, which combines NLP and ML techniques, to enhance AERD diagnosis in practice. On top of prior work [3], we used NLP with a potential feature—urine leukotriene E4 levels from EHR—which have been shown to aid in AERD diagnosis [15]. Leveraging NLP and ML techniques in practice has the potential to reduce diagnostic delays for AERD and improve the health of patients.

## Abbreviations

AERD: aspirin-exacerbated respiratory disease
AUC: area under the receiver operating characteristic curve
EHR: electronic health record
ICD: International Classification of Diseases
ML: machine learning
NLP: natural language processing
NSAID: nonsteroidal anti-inflammatory drug
